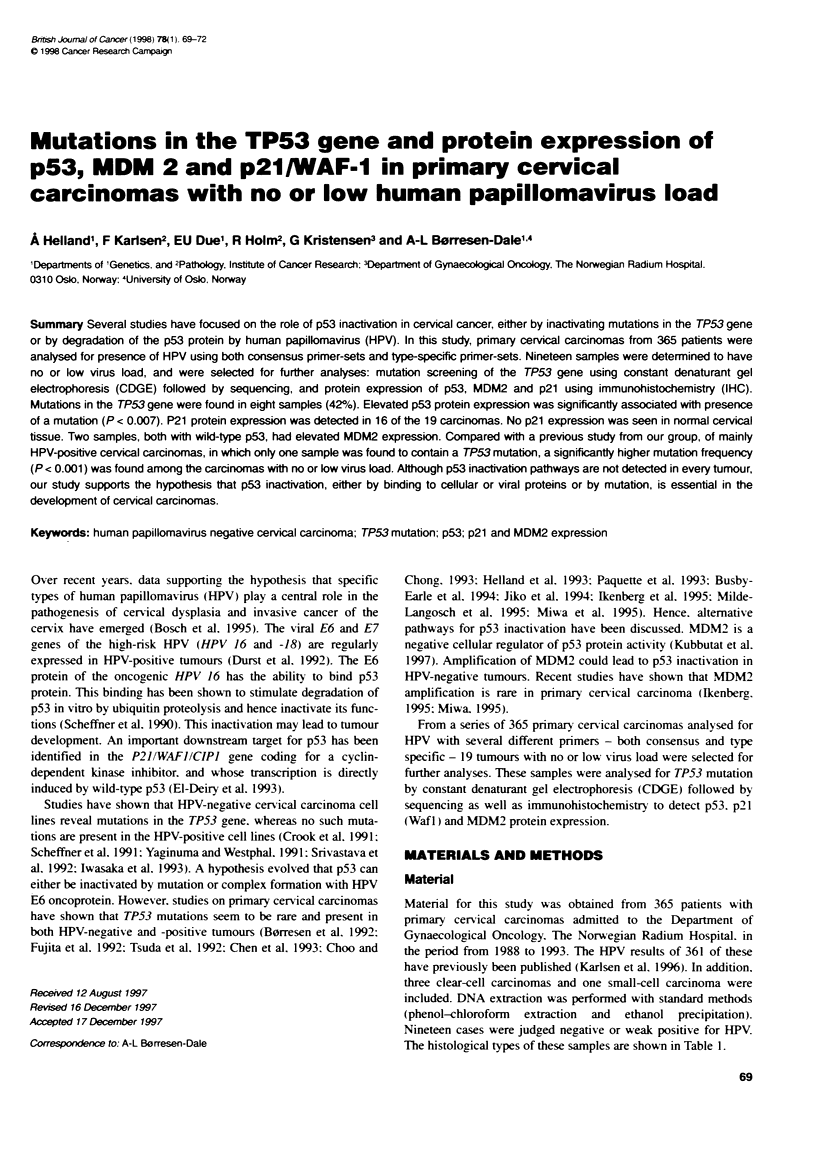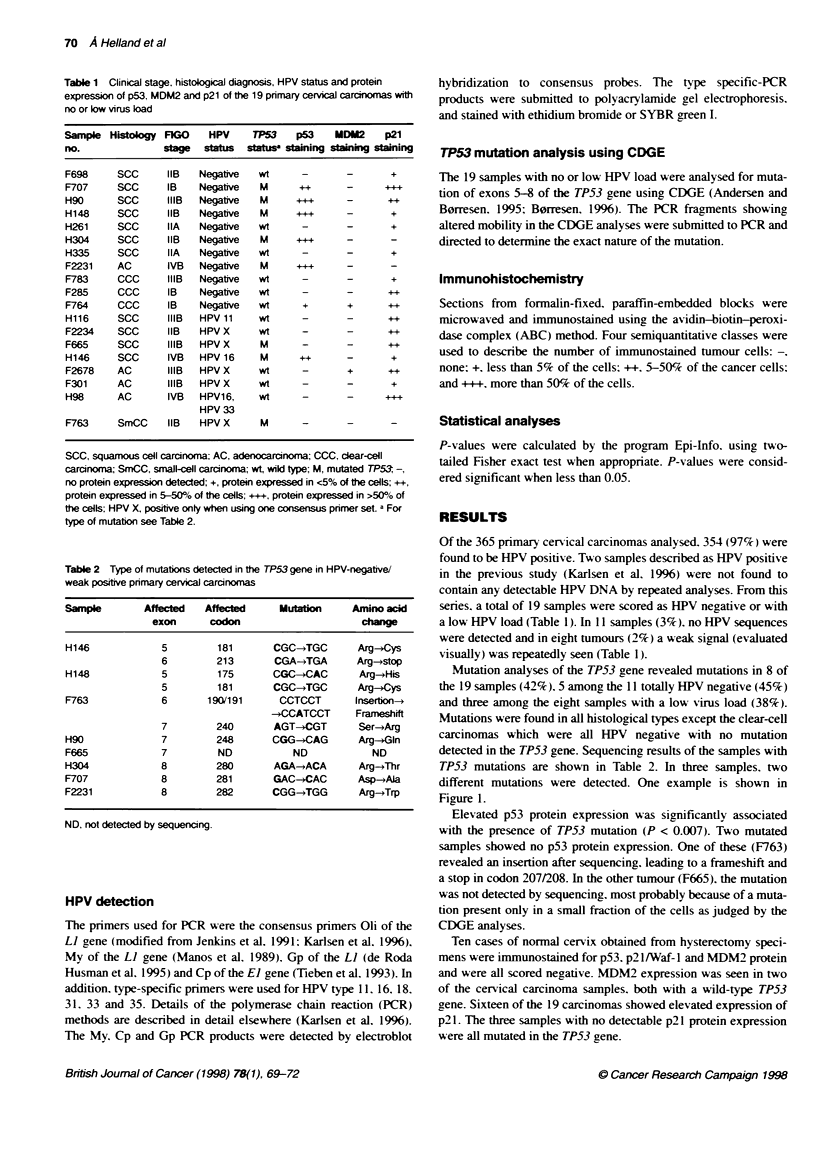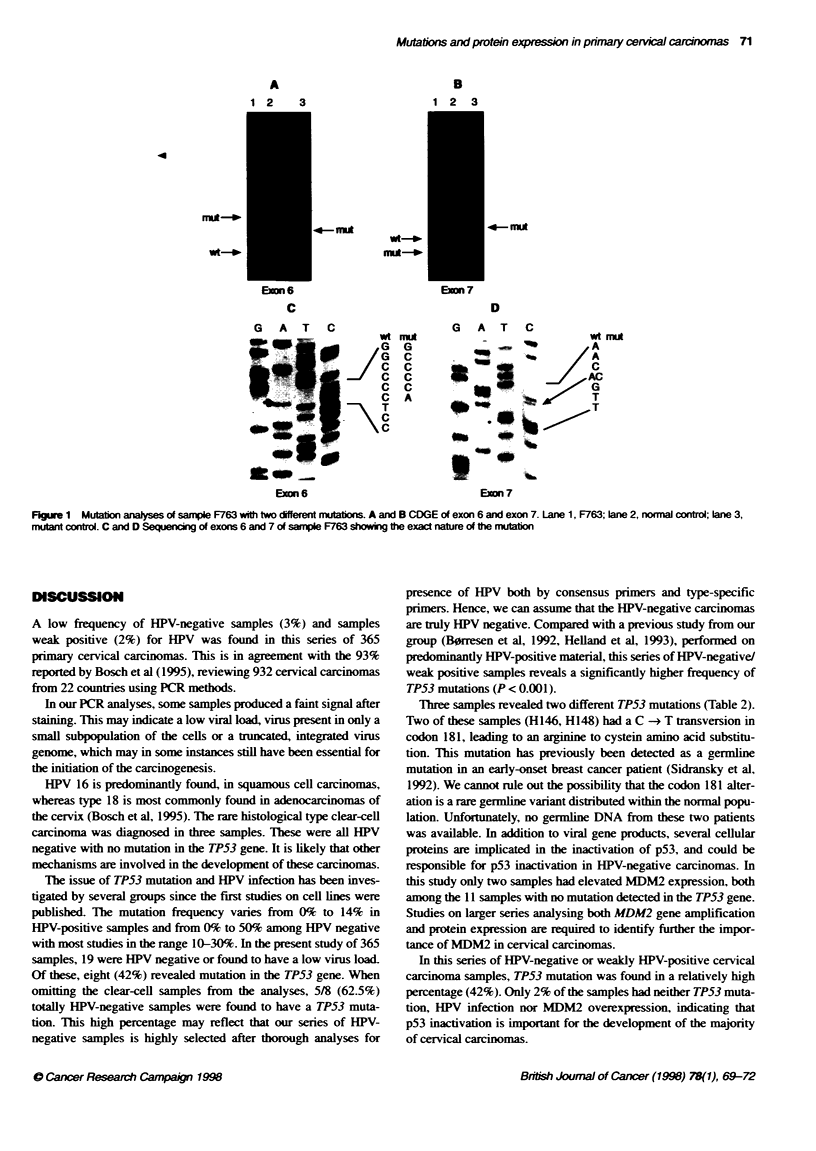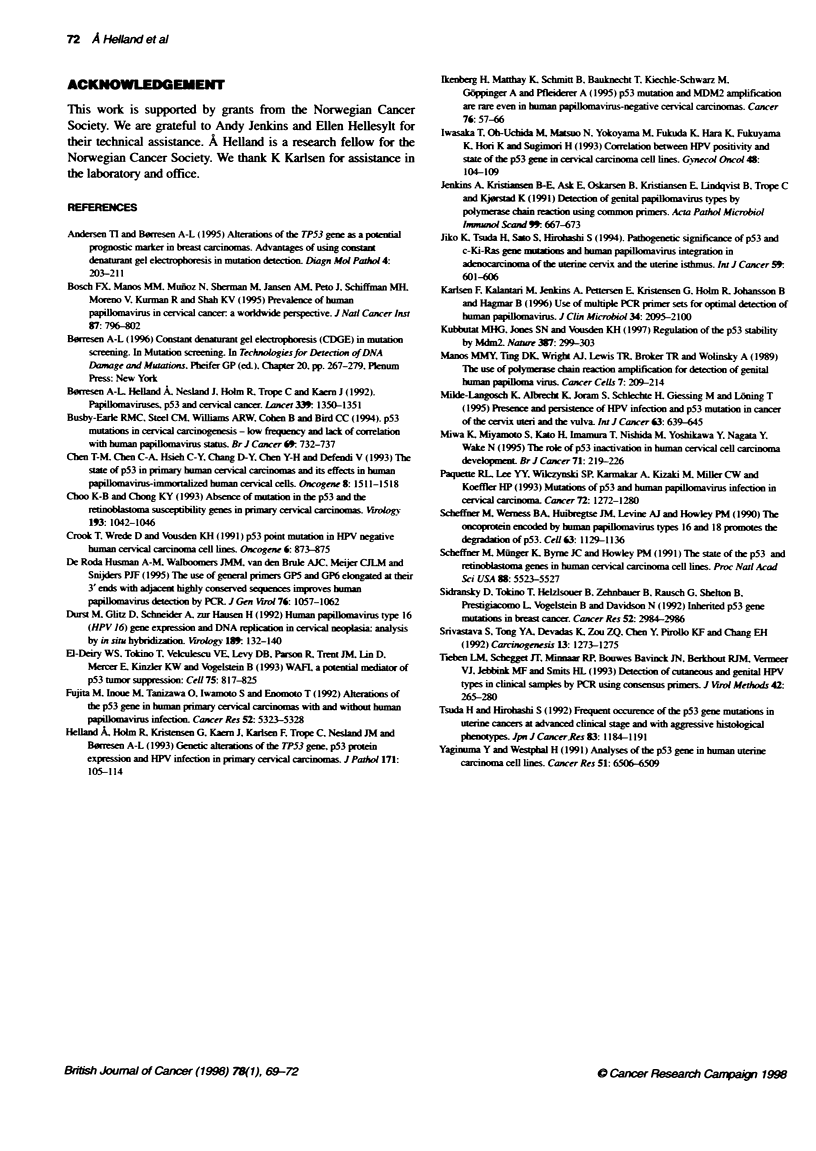# Mutations in the TP53 gene and protein expression of p53, MDM 2 and p21/WAF-1 in primary cervical carcinomas with no or low human papillomavirus load.

**DOI:** 10.1038/bjc.1998.444

**Published:** 1998-07

**Authors:** A. Helland, F. Karlsen, E. U. Due, R. Holm, G. Kristensen, A. l. Børresen-Dale

**Affiliations:** Department of Genetics, Institute of Cancer Research, Oslo, Norway.

## Abstract

**Images:**


British Journal of Cancer (1998) 78(1), 69–72
© 1998 Cancer Research Campaign

### Mutations in the TP53 gene and protein expression of p53, MDM 2 and p21/WAF-1 in primary cervical carcinomas with no or low human papillomavirus load

**Å Helland[1], F Karlsen[2], EU Due[1], R Holm[2], G Kristensen[3] and A-L Børresen-Dale[1,4]**

[1]Departments of [1]Genetics, and [2]Pathology, Institute of Cancer Research; [3]Department of Gynaecological Oncology, The Norwegian Radium Hospital, 0310 Oslo, Norway; [4]University of Oslo, Norway

**Summary** Several studies have focused on the role of p53 inactivation in cervical cancer, either by inactivating mutations in the *TP53* gene or by degradation of the p53 protein by human papillomavirus (HPV). In this study, primary cervical carcinomas from 365 patients were analysed for presence of HPV using both consensus primer-sets and type-specific primer-sets. Nineteen samples were determined to have no or low virus load, and were selected for further analyses: mutation screening of the *TP53* gene using constant denaturant gel electrophoresis (CDGE) followed by sequencing, and protein expression of p53, MDM2 and p21 using immunohistochemistry (IHC). Mutations in the *TP53* gene were found in eight samples (42%). Elevated p53 protein expression was significantly associated with presence of a mutation ($P < 0.007$). P21 protein expression was detected in 16 of the 19 carcinomas. No p21 expression was seen in normal cervical tissue. Two samples, both with wild-type p53, had elevated MDM2 expression. Compared with a previous study from our group, of mainly HPV-positive cervical carcinomas, in which only one sample was found to contain a *TP53* mutation, a significantly higher mutation frequency ($P < 0.001$) was found among the carcinomas with no or low virus load. Although p53 inactivation pathways are not detected in every tumour, our study supports the hypothesis that p53 inactivation, either by binding to cellular or viral proteins or by mutation, is essential in the development of cervical carcinomas.

**Keywords:** human papillomavirus negative cervical carcinoma; *TP53* mutation; p53; p21 and MDM2 expression

Over recent years, data supporting the hypothesis that specific types of human papillomavirus (HPV) play a central role in the pathogenesis of cervical dysplasia and invasive cancer of the cervix have emerged (Bosch et al, 1995). The viral *E6* and *E7* genes of the high-risk HPV (*HPV 16* and *-18*) are regularly expressed in HPV-positive tumours (Durst et al, 1992). The E6 protein of the oncogenic *HPV 16* has the ability to bind p53 protein. This binding has been shown to stimulate degradation of p53 in vitro by ubiquitin proteolysis and hence inactivate its functions (Scheffner et al, 1990). This inactivation may lead to tumour development. An important downstream target for p53 has been identified in the *P21/WAF1/CIP1* gene coding for a cyclin-dependent kinase inhibitor, and whose transcription is directly induced by wild-type p53 (El-Deiry et al, 1993).

Studies have shown that HPV-negative cervical carcinoma cell lines reveal mutations in the *TP53* gene, whereas no such mutations are present in the HPV-positive cell lines (Crook et al, 1991; Scheffner et al, 1991; Yaginuma and Westphal, 1991; Srivastava et al, 1992; Iwasaka et al, 1993). A hypothesis evolved that p53 can either be inactivated by mutation or complex formation with HPV E6 oncoprotein. However, studies on primary cervical carcinomas have shown that *TP53* mutations seem to be rare and present in both HPV-negative and -positive tumours (Børresen et al, 1992; Fujita et al, 1992; Tsuda et al, 1992; Chen et al, 1993; Choo and Chong, 1993; Helland et al, 1993; Paquette et al, 1993; Busby-Earle et al, 1994; Jiko et al, 1994; Ikenberg et al, 1995; Milde-Langosch et al, 1995; Miwa et al, 1995). Hence, alternative pathways for p53 inactivation have been discussed. MDM2 is a negative cellular regulator of p53 protein activity (Kubbutat et al, 1997). Amplification of MDM2 could lead to p53 inactivation in HPV-negative tumours. Recent studies have shown that MDM2 amplification is rare in primary cervical carcinoma (Ikenberg, 1995; Miwa, 1995).

*Received 12 August 1997*
*Revised 16 December 1997*
*Accepted 17 December 1997*

*Correspondence to:* A-L Børresen-Dale

From a series of 365 primary cervical carcinomas analysed for HPV with several different primers – both consensus and type specific – 19 tumours with no or low virus load were selected for further analyses. These samples were analysed for *TP53* mutation by constant denaturant gel electrophoresis (CDGE) followed by sequencing as well as immunohistochemistry to detect p53, p21 (Waf1) and MDM2 protein expression.

### MATERIALS AND METHODS

### Material

Material for this study was obtained from 365 patients with primary cervical carcinomas admitted to the Department of Gynaecological Oncology, The Norwegian Radium Hospital, in the period from 1988 to 1993. The HPV results of 361 of these have previously been published (Karlsen et al, 1996). In addition, three clear-cell carcinomas and one small-cell carcinoma were included. DNA extraction was performed with standard methods (phenol–chloroform extraction and ethanol precipitation). Nineteen cases were judged negative or weak positive for HPV. The histological types of these samples are shown in Table 1.

Table 1 Clinical stage, histological diagnosis, HPV status and protein expression of p53, MDM2 and p21 of the 19 primary cervical carcinomas with no or low virus load

| Sample no. | Histology | FIGO stage | HPV status | *TP53* status[a] | p53 staining | MDM2 staining | p21 staining |
|---|---|---|---|---|---|---|---|
| F698 | SCC | IIB | Negative | wt | – | – | + |
| F707 | SCC | IB | Negative | M | ++ | – | +++ |
| H90 | SCC | IIIB | Negative | M | +++ | – | ++ |
| H148 | SCC | IIB | Negative | M | +++ | – | + |
| H261 | SCC | IIA | Negative | wt | – | – | + |
| H304 | SCC | IIB | Negative | M | +++ | – | – |
| H335 | SCC | IIA | Negative | wt | – | – | + |
| F2231 | AC | IVB | Negative | M | +++ | – | – |
| F783 | CCC | IIIB | Negative | wt | – | – | + |
| F285 | CCC | IB | Negative | wt | – | – | ++ |
| F764 | CCC | IB | Negative | wt | + | + | ++ |
| H116 | SCC | IIIB | HPV 11 | wt | – | – | ++ |
| F2234 | SCC | IIB | HPV X | wt | – | – | ++ |
| F665 | SCC | IIIB | HPV X | M | – | – | ++ |
| H146 | SCC | IVB | HPV 16 | M | ++ | – | + |
| F2678 | AC | IIIB | HPV X | wt | – | + | ++ |
| F301 | AC | IIIB | HPV X | wt | – | – | + |
| H98 | AC | IVB | HPV16, HPV 33 | wt | – | – | +++ |
| F763 | SmCC | IIB | HPV X | M | – | – | – |

SCC, squamous cell carcinoma; AC, adenocarcinoma; CCC, clear-cell carcinoma; SmCC, small-cell carcinoma; wt, wild type; M, mutated *TP53*; –, no protein expression detected; +, protein expressed in <5% of the cells; ++, protein expressed in 5–50% of the cells; +++, protein expressed in >50% of the cells; HPV X, positive only when using one consensus primer set. [a] For type of mutation see Table 2.

Table 2 Type of mutations detected in the *TP53* gene in HPV-negative/weak positive primary cervical carcinomas

| Sample | Affected exon | Affected codon | Mutation | Amino acid change |
|---|---|---|---|---|
| H146 | 5 | 181 | CGC→TGC | Arg→Cys |
| | 6 | 213 | CGA→TGA | Arg→stop |
| H148 | 5 | 175 | CGC→CAC | Arg→His |
| | 5 | 181 | CGC→TGC | Arg→Cys |
| F763 | 6 | 190/191 | CCTCCT →CCATCCT | Insertion→ Frameshift |
| | 7 | 240 | AGT→CGT | Ser→Arg |
| H90 | 7 | 248 | CGG→CAG | Arg→Gln |
| F665 | 7 | ND | ND | ND |
| H304 | 8 | 280 | AGA→ACA | Arg→Thr |
| F707 | 8 | 281 | GAC→CAC | Asp→Ala |
| F2231 | 8 | 282 | CGG→TGG | Arg→Trp |

ND, not detected by sequencing.

### HPV detection

The primers used for PCR were the consensus primers Oli of the *L1* gene (modified from Jenkins et al, 1991; Karlsen et al, 1996), My of the *L1* gene (Manos et al, 1989), Gp of the *L1* (de Roda Husman et al, 1995) and Cp of the *E1* gene (Tieben et al, 1993). In addition, type-specific primers were used for HPV type 11, 16, 18, 31, 33 and 35. Details of the polymerase chain reaction (PCR) methods are described in detail elsewhere (Karlsen et al, 1996). The My, Cp and Gp PCR products were detected by electroblot hybridization to consensus probes. The type specific-PCR products were submitted to polyacrylamide gel electrophoresis, and stained with ethidium bromide or SYBR green I.

### *TP53* mutation analysis using CDGE

The 19 samples with no or low HPV load were analysed for mutation of exons 5–8 of the *TP53* gene using CDGE (Andersen and Børresen, 1995; Børresen, 1996). The PCR fragments showing altered mobility in the CDGE analyses were submitted to PCR and directed to determine the exact nature of the mutation.

### Immunohistochemistry

Sections from formalin-fixed, paraffin-embedded blocks were microwaved and immunostained using the avidin–biotin–peroxidase complex (ABC) method. Four semiquantitative classes were used to describe the number of immunostained tumour cells: –, none; +, less than 5% of the cells; ++, 5–50% of the cancer cells; and +++, more than 50% of the cells.

### Statistical analyses

*P*-values were calculated by the program Epi-Info, using two-tailed Fisher exact test when appropriate. *P*-values were considered significant when less than 0.05.

### RESULTS

Of the 365 primary cervical carcinomas analysed, 354 (97%) were found to be HPV positive. Two samples described as HPV positive in the previous study (Karlsen et al, 1996) were not found to contain any detectable HPV DNA by repeated analyses. From this series, a total of 19 samples were scored as HPV negative or with a low HPV load (Table 1). In 11 samples (3%), no HPV sequences were detected and in eight tumours (2%) a weak signal (evaluated visually) was repeatedly seen (Table 1).

Mutation analyses of the *TP53* gene revealed mutations in 8 of the 19 samples (42%), 5 among the 11 totally HPV negative (45%) and three among the eight samples with a low virus load (38%). Mutations were found in all histological types except the clear-cell carcinomas which were all HPV negative with no mutation detected in the *TP53* gene. Sequencing results of the samples with *TP53* mutations are shown in Table 2. In three samples, two different mutations were detected. One example is shown in Figure 1.

Elevated p53 protein expression was significantly associated with the presence of *TP53* mutation ($P < 0.007$). Two mutated samples showed no p53 protein expression. One of these (F763) revealed an insertion after sequencing, leading to a frameshift and a stop in codon 207/208. In the other tumour (F665), the mutation was not detected by sequencing, most probably because of a mutation present only in a small fraction of the cells as judged by the CDGE analyses.

Ten cases of normal cervix obtained from hysterectomy specimens were immunostained for p53, p21/Waf-1 and MDM2 protein and were all scored negative. MDM2 expression was seen in two of the cervical carcinoma samples, both with a wild-type *TP53* gene. Sixteen of the 19 carcinomas showed elevated expression of p21. The three samples with no detectable p21 protein expression were all mutated in the *TP53* gene.

© Cancer Research Campaign 1998

**Figure 1** Mutation analyses of sample F763 with two different mutations. **A** and **B** CDGE of exon 6 and exon 7. Lane 1, F763; lane 2, normal control; lane 3, mutant control. **C** and **D** Sequencing of exons 6 and 7 of sample F763 showing the exact nature of the mutation

### DISCUSSION

A low frequency of HPV-negative samples (3%) and samples weak positive (2%) for HPV was found in this series of 365 primary cervical carcinomas. This is in agreement with the 93% reported by Bosch et al (1995), reviewing 932 cervical carcinomas from 22 countries using PCR methods.

In our PCR analyses, some samples produced a faint signal after staining. This may indicate a low viral load, virus present in only a small subpopulation of the cells or a truncated, integrated virus genome, which may in some instances still have been essential for the initiation of the carcinogenesis.

HPV 16 is predominantly found, in squamous cell carcinomas, whereas type 18 is most commonly found in adenocarcinomas of the cervix (Bosch et al, 1995). The rare histological type clear-cell carcinoma was diagnosed in three samples. These were all HPV negative with no mutation in the *TP53* gene. It is likely that other mechanisms are involved in the development of these carcinomas.

The issue of *TP53* mutation and HPV infection has been investigated by several groups since the first studies on cell lines were published. The mutation frequency varies from 0% to 14% in HPV-positive samples and from 0% to 50% among HPV negative with most studies in the range 10–30%. In the present study of 365 samples, 19 were HPV negative or found to have a low virus load. Of these, eight (42%) revealed mutation in the *TP53* gene. When omitting the clear-cell samples from the analyses, 5/8 (62.5%) totally HPV-negative samples were found to have a *TP53* mutation. This high percentage may reflect that our series of HPV-negative samples is highly selected after thorough analyses for presence of HPV both by consensus primers and type-specific primers. Hence, we can assume that the HPV-negative carcinomas are truly HPV negative. Compared with a previous study from our group (Børresen et al, 1992, Helland et al, 1993), performed on predominantly HPV-positive material, this series of HPV-negative/weak positive samples reveals a significantly higher frequency of *TP53* mutations ($P < 0.001$).

Three samples revealed two different *TP53* mutations (Table 2). Two of these samples (H146, H148) had a C → T transversion in codon 181, leading to an arginine to cystein amino acid substitution. This mutation has previously been detected as a germline mutation in an early-onset breast cancer patient (Sidransky et al, 1992). We cannot rule out the possibility that the codon 181 alteration is a rare germline variant distributed within the normal population. Unfortunately, no germline DNA from these two patients was available. In addition to viral gene products, several cellular proteins are implicated in the inactivation of p53, and could be responsible for p53 inactivation in HPV-negative carcinomas. In this study only two samples had elevated MDM2 expression, both among the 11 samples with no mutation detected in the *TP53* gene. Studies on larger series analysing both *MDM2* gene amplification and protein expression are required to identify further the importance of MDM2 in cervical carcinomas.

In this series of HPV-negative or weakly HPV-positive cervical carcinoma samples, *TP53* mutation was found in a relatively high percentage (42%). Only 2% of the samples had neither *TP53* mutation, HPV infection nor MDM2 overexpression, indicating that p53 inactivation is important for the development of the majority of cervical carcinomas.

© Cancer Research Campaign 1998 

### ACKNOWLEDGEMENT

This work is supported by grants from the Norwegian Cancer Society. We are grateful to Andy Jenkins and Ellen Hellesylt for their technical assistance. Å Helland is a research fellow for the Norwegian Cancer Society. We thank K Karlsen for assistance in the laboratory and office.

### REFERENCES

Andersen TI and Børresen A-L (1995) Alterations of the *TP53* gene as a potential prognostic marker in breast carcinomas. Advantages of using constant denaturant gel electrophoresis in mutation detection. *Diagn Mol Pathol* 4: 203–211

Bosch FX, Manos MM, Muñoz N, Sherman M, Jansen AM, Peto J, Schiffman MH, Moreno V, Kurman R and Shah KV (1995) Prevalence of human papillomavirus in cervical cancer: a worldwide perspective. *J Natl Cancer Inst* 87: 796–802

Børresen A-L (1996) Constant denaturant gel electrophoresis (CDGE) in mutation screening. In Mutation screening. In *Technologies for Detection of DNA Damage and Mutations*, Pheifer GP (ed.), Chapter 20, pp. 267–279, Plenum Press: New York

Børresen A-L, Helland Å, Nesland J, Holm R, Trope C and Kaern J (1992). Papillomaviruses, p53 and cervical cancer. *Lancet* 339: 1350–1351

Busby-Earle RMC, Steel CM, Williams ARW, Cohen B and Bird CC (1994). p53 mutations in cervical carcinogenesis – low frequency and lack of correlation with human papillomavirus status. *Br J Cancer* 69: 732–737

Chen T-M, Chen C-A, Hsieh C-Y, Chang D-Y, Chen Y-H and Defendi V (1993) The state of p53 in primary human cervical carcinomas and its effects in human papillomavirus-immortalized human cervical cells. *Oncogene* 8: 1511–1518

Choo K-B and Chong KY (1993) Absence of mutation in the p53 and the retinoblastoma susceptibility genes in primary cervical carcinomas. *Virology* 193: 1042–1046

Crook T, Wrede D and Vousden KH (1991) p53 point mutation in HPV negative human cervical carcinoma cell lines. *Oncogene* 6: 873–875

De Roda Husman A-M, Walboomers JMM, van den Brule AJC, Meijer CJLM and Snijders PJF (1995) The use of general primers GP5 and GP6 elongated at their 3′ ends with adjacent highly conserved sequences improves human papillomavirus detection by PCR. *J Gen Virol* 76: 1057–1062

Durst M, Glitz D, Schneider A, zur Hausen H (1992) Human papillomavirus type 16 (*HPV 16*) gene expression and DNA replication in cervical neoplasia: analysis by *in situ* hybridization. *Virology* 189: 132–140

El-Deiry WS, Tokino T, Velculescu VE, Levy DB, Parson R, Trent JM, Lin D, Mercer E, Kinzler KW and Vogelstein B (1993) WAF1, a potential mediator of p53 tumor suppression: *Cell* 75: 817–825

Fujita M, Inoue M, Tanizawa O, Iwamoto S and Enomoto T (1992) Alterations of the p53 gene in human primary cervical carcinomas with and without human papillomavirus infection. *Cancer Res* 52: 5323–5328

Helland Å, Holm R, Kristensen G, Kaern J, Karlsen F, Trope C, Nesland JM and Børresen A-L (1993) Genetic alterations of the *TP53* gene, p53 protein expression and HPV infection in primary cervical carcinomas. *J Pathol* 171: 105–114

Ikenberg H, Matthay K, Schmitt B, Bauknecht T, Kiechle-Schwarz M, Göppinger A and Pfleiderer A (1995) p53 mutation and MDM2 amplification are rare even in human papillomavirus-negative cervical carcinomas. *Cancer* 76: 57–66

Iwasaka T, Oh-Uchida M, Matsuo N, Yokoyama M, Fukuda K, Hara K, Fukuyama K, Hori K and Sugimori H (1993) Correlation between HPV positivity and state of the p53 gene in cervical carcinoma cell lines. *Gynecol Oncol* 48: 104–109

Jenkins A, Kristiansen B-E, Ask E, Oskarsen B, Kristiansen E, Lindqvist B, Trope C and Kjørstad K (1991) Detection of genital papillomavirus types by polymerase chain reaction using common primers. *Acta Pathol Microbiol Immunol Scand* 99: 667–673

Jiko K, Tsuda H, Sato S, Hirohashi S (1994). Pathogenetic significance of p53 and c-Ki-Ras gene mutations and human papillomavirus integration in adenocarcinoma of the uterine cervix and the uterine isthmus. *Int J Cancer* 59: 601–606

Karlsen F, Kalantari M, Jenkins A, Pettersen E, Kristensen G, Holm R, Johansson B and Hagmar B (1996) Use of multiple PCR primer sets for optimal detection of human papillomavirus. *J Clin Microbiol* 34: 2095–2100

Kubbutat MHG, Jones SN and Vousden KH (1997) Regulation of the p53 stability by Mdm2. *Nature* 387: 299–303

Manos MMY, Ting DK, Wright AJ, Lewis TR, Broker TR and Wolinsky A (1989) The use of polymerase chain reaction amplification for detection of genital human papilloma virus. *Cancer Cells* 7: 209–214

Milde-Langosch K, Albrecht K, Joram S, Schlechte H, Giessing M and Löning T (1995) Presence and persistence of HPV infection and p53 mutation in cancer of the cervix uteri and the vulva. *Int J Cancer* 63: 639–645

Miwa K, Miyamoto S, Kato H, Imamura T, Nishida M, Yoshikawa Y, Nagata Y, Wake N (1995) The role of p53 inactivation in human cervical cell carcinoma development. *Br J Cancer* 71: 219–226

Paquette RL, Lee YY, Wilczynski SP, Karmakar A, Kizaki M, Miller CW and Koeffler HP (1993) Mutations of p53 and human papillomavirus infection in cervical carcinoma. *Cancer* 72: 1272–1280

Scheffner M, Werness BA, Huibregtse JM, Levine AJ and Howley PM (1990) The oncoprotein encoded by human papillomavirus types 16 and 18 promotes the degradation of p53. *Cell* 63: 1129–1136

Scheffner M, Münger K, Byrne JC and Howley PM (1991) The state of the p53 and retinoblastoma genes in human cervical carcinoma cell lines. *Proc Natl Acad Sci USA* 88: 5523–5527

Sidransky D, Tokino T, Helzlsouer B, Zehnbauer B, Rausch G, Shelton B, Prestigiacomo L, Vogelstein B and Davidson N (1992) Inherited p53 gene mutations in breast cancer. *Cancer Res* 52: 2984–2986

Srivastava S, Tong YA, Devadas K, Zou ZQ, Chen Y, Pirollo KF and Chang EH (1992) *Carcinogenesis* 13: 1273–1275

Tieben LM, Schegget JT, Minnaar RP, Bouwes Bavinck JN, Berkhout RJM, Vermeer VJ, Jebbink MF and Smits HL (1993) Detection of cutaneous and genital HPV types in clinical samples by PCR using consensus primers. *J Virol Methods* 42: 265–280

Tsuda H and Hirohashi S (1992) Frequent occurence of the p53 gene mutations in uterine cancers at advanced clinical stage and with aggressive histological phenotypes. *Jpn J Cancer Res* 83: 1184–1191

Yaginuma Y and Westphal H (1991) Analyses of the p53 gene in human uterine carcinoma cell lines. *Cancer Res* 51: 6506–6509

© Cancer Research Campaign 1998